# Constructing Temporally Extended Actions through Incremental Community Detection

**DOI:** 10.1155/2018/2085721

**Published:** 2018-04-23

**Authors:** Xiao Xu, Mei Yang, Ge Li

**Affiliations:** College of System Engineering, National University of Defense Technology, Changsha, Hunan 410073, China

## Abstract

Hierarchical reinforcement learning works on temporally extended actions or skills to facilitate learning. How to automatically form such abstraction is challenging, and many efforts tackle this issue in the options framework. While various approaches exist to construct options from different perspectives, few of them concentrate on options' adaptability during learning. This paper presents an algorithm to create options and enhance their quality online. Both aspects operate on detected communities of the learning environment's state transition graph. We first construct options from initial samples as the basis of online learning. Then a rule-based community revision algorithm is proposed to update graph partitions, based on which existing options can be continuously tuned. Experimental results in two problems indicate that options from initial samples may perform poorly in more complex environments, and our presented strategy can effectively improve options and get better results compared with flat reinforcement learning.

## 1. Introduction

Reinforcement learning (RL) is a machine learning branch where an agent learns to optimize its behavior by trial-and-error interaction with its environment. Traditional RL researches suffer the inability of use in complex practical problems due to the so-called “Curse-of-Dimensionality,” that is, the exponential growth of memory requirements with the number of state variables. Hierarchical Reinforcement Learning (HRL) aims to reduce the dimensionality through decomposing the RL problem into several subproblems. As solving small-scale subproblems would be simpler than solving the entire one, HRL is expected to be more efficient than flat RL. In the HRL research community, three main frameworks, HAM [1], options framework [2], and MAX-Q [3], provide different paradigms of problem hierarchies and learning methodologies. These all make HRL work on temporally extended actions or skills. Generally HRL requires domain knowledge to define such abstraction, which may function only for specific problems. How to automatically form useful abstractions, or skill acquisition, is an attractive issue.

To the best of our knowledge, most studies on this topic adopt the options framework [4–6]. Then the skill acquisition falls in automatic option construction. Though existing approaches solve this from different perspectives, one thing in common is that they require data sampled from the environment. In some complex environments this sampled experience may be deficient in describing the actual transition dynamics of the environment. These previously created options may get unadaptable to the environment, thus even leading to poor performance in HRL. For such cases there is need to online improve the quality of individual options.

This paper targets two problems, that is, how to create options and how to optimize options during learning. Our approach is to operate on the state transition graph of the learning environment. In the graph states are individual nodes and connecting edges denote states transitions. We first divide the sampled graph as communities, from which options are constructed. Community is a concept in the network science field, representing a cluster of strongly connected states. This paper employs Louvain algorithm [7] for community detection. The generated option set acts as the base for online learning. We present a rule-based community revision algorithm, adding newly collected states and transitions to previous communities. Option improvement is then performed based on these updated communities. Our approach is evaluated in two environments, that is, four-room grid world and small-scale Pac-Man world. The former is a benchmark problem for testing option generation algorithms, in which the effectiveness of learning options from Louvain detected communities is tested. The latter is more complex and uncertain, which is suitable for demonstrating the performance of the presented incremental-option improvement algorithm. Comparative results show that, in the four-room environment, options constructed from communities can accelerate the convergence speed than learning from primitive actions. In the Pac-Man environment, two scenarios with different types of the ghost agent are set. One follows a fixed strategy while the other makes random moves. Results suggest that options from initial samples perform poorly in the more complex scenario, while the presented incremental-option improvement can help adapt the existing option set and obtain better results compared with flat RL.

The remainder of this paper is organized as follows: In Section 2 we describe some basic ideas of RL and the options framework.  Section 3 shows some related works on option construction. In Section 4 we illustrate the main approach of creating options from communities.  Section 5 gives the detailed algorithm on incremental community revision and how to learn from these evolving communities.  Section 6 demonstrates experiments and result analysis. Finally we discuss our implementation and draw conclusions in Section 7.

## 2. Preliminaries

### 2.1. Reinforcement Learning

The RL environment is typically formalized as a Markov Decision Process (MDP) [8] that can be described as a 5-element tuple (*𝒮*, *𝒜*, *P*, *R*, *γ*), where *𝒮* is a finite set of states of the learning environment; *𝒜*_*s*_ is the available action set in state *s*; *P* : *𝒮* × *𝒜* × *𝒮* → [0,1] describes the state transition dynamics; *R* : *𝒮* × *𝒜* → *ℝ* represents the reward function for each state transition; and the discount factor *γ* ∈ [0,1] is to balance the importance of short-term and long-term reward. At each step of time *t*, an agent in state *s*_*t*_ ∈ *𝒮* selects an available action *a*_*t*_ ∈ *𝒜*_*s*_; then at next step it moves to *s*_*t*+1_ with the probability *P*(*s*_*t*_, *a*_*t*_, *s*_*t*+1_) and obtains the reward *r*_*t*+1_ = *R*(*s*_*t*_, *a*_*t*_). A policy *π* : *𝒮* → *𝒜* defines which action to choose under a certain state. It is associated with the action-value function *Q*_*π*_(*s*, *a*) = *𝔼*_*π*_[∑_*k*=0_^*∞*^*γ*^*k*^*r*_*t*+*k*+1_∣*s*_*t*_ = *s*, *a*_*t*_ = *a*] indicating the expected reward from *s* after taking *a* and thereafter following *π*. The aim of RL is to find an optimal policy *π*^*∗*^ that can reach the maximal expected reward, corresponding to the optimal action-value function *Q*^*∗*^ = max_*π*_⁡*Q*_*π*_(*s*, *a*).

RL algorithms can be divided as two categories, that is, model-based and model-free, according to whether they attempt to model the environment. *Q*-learning [9], one of the most commonly used RL algorithms, is a model-free type. In each learning step, the agent experiences the transition (*s*_*t*_, *a*_*t*_, *s*_*t*+1_, *r*_*t*+1_), and then the *Q* function is updated as follows:

(1) $$\begin{array}{c} Q\left(\;s_{t},a_{t}\;\right)\overset{\alpha }{⟵}r_{t+1}+\gamma \;\underset{a}{\max}\;Q\left(\;s_{t+1},a\;\right)-Q\left(\;s_{t},a_{t}\;\right), \end{array}$$

where *α* ∈ (0,1] is the learning rate. *Q*-learning has been shown to converge to *Q*^*∗*^ under standard stochastic approximation assumptions.

### 2.2. Options Framework

The MDP model assumes that an action lasts for a single time unit. In large state space problems, hierarchical abstraction has proven to be able to increase the RL efficiency. Options [2], built on these one-step actions, are formed as temporally extended courses of actions. An option is defined as a triple *o* = 〈*ℐ*, *π*_*o*_, *β*〉, where

- *ℐ* ∈ *𝒮* is the* initiation set*; that is, *o* is applicable in *s* iff *s* ∈ *ℐ*;
- *π*_*o*_ : *𝒮* → *𝒜* defines the* option policy*;
- *β* : *𝒮* → [0,1] specifies the* termination condition* while executing *o*.

With this definition, an atom action *a* can also be viewed as a* primitive option* with the initiation set *ℐ* = {*s* : *a* ∈ *𝒜*_*s*_}, the local policy *π*_*o*_(*s*) = *a*, ∀*s* ∈ *ℐ*, and the one-step termination condition *β*(*s*) = 1, ∀*s* ∈ *𝒮*. Thus the option based RL agent can choose among atom actions as well as higher level skills.

The MDP model with a set of options *𝒪* is formed as a Semi-Markov Decision Process (SMDP). When the learning agent chooses an option to perform, it follows the option policy for several steps until the termination condition is satisfied. *Q*-learning under SMDP, which is also referred to as Option-to-Option Learning, updates the option value function after the option has terminated. Specifically, the rule is as

(2) Qst,ot⟵α∑k=0n−1γkrt+k+1+γn maxo∈O⁡ Qst+n,o−Qst,ot,

where *s*_*t*_ is the starting state of the option *o*_*t*_, *n* is the number of steps where *o*_*t*_ is taken from *s*_*t*_ to its ending, and *s*_*t*+*n*_ is the state that *o*_*t*_ terminates at.

The main drawback of Option-to-Option Learning is that it needs to execute an option to completion before learning its value, thus requiring a significant amount of experience to reach convergence for every option. On the other hand, intraoption learning [10] can take advantage of one-step option execution for all related options, which leads to potentially more efficient learning. In detail, an experience fragment (*s*_*t*_, *a*_*t*_, *s*_*t*+1_, *r*_*t*+1_) can be utilized for all consistent options, which would have taken *a*_*t*_ in *s*_*t*_, to update the value estimation. Such one-step intraoption value learning is expressed as

(3) $$\begin{array}{c} Q\left(\;s_{t},o\;\right)\overset{\alpha }{⟵}r_{t+1}+\gamma U\left(\;s_{t+1},o\;\right)-Q\left(\;s_{t},o\;\right), \end{array}$$

where

(4) $$\begin{array}{c} U\left(\;s,o\;\right)=\left(\;1-\beta \left(\;s\;\right)\;\right)Q\left(\;s,o\;\right)+\beta \left(\;s\;\right)\underset{o^{\prime }\in O}{\max}\;Q\left(\;s,o^{\prime }\;\right). \end{array}$$

This update rule takes place after the one-step transition and is applied by options which are consistent with the policy *π*(*s*_*t*_) = *a*_*t*_.

The options framework illustrates how defined options are utilized by the learning agent. What we concern here is how to form useful options, having their *ℐ*, *π*_*o*_, and *β* automatically generated.

## 3. Literature Review

Automated option construction has been an active research area and various approaches have been proposed. These efforts commonly work on the basis of sampled experience. With collected states and transitions, a general process is to identify useful* subgoals* and then compose options using them. The term of subgoals defines what states the options need to achieve. They act as the basis to divide the original problem. Based on the difference in finding subgoals, most existing works can be categorized as two main branches: the sampling trajectory based approach and the graphical approach.

The sampling trajectory based approach tries to analyze history experience from a statistical perspective. For instance, the diverse density algorithm [4] specifies subgoals as regions where the agent passes more frequently on successful trajectories and not on unsuccessful ones. Here the successful trajectory is defined where it can start from any state and finally end at the expected goal state. The relative novelty [11] assumes that subgoals can lead the agent to access a new region from highly visited regions. The Local Roots algorithm [12] considers that subgoals should be junctions of shortcut paths from each state to the goal state. This approach online constructs a sequence tree from collected successful trajectories and takes the one with the local maximum* root factor* measure as subgoal. Another method presented in [13] employs the ant colony optimization to construct options. In its context subgoals are specified by monitoring the variance in pheromone values of related transitions.

The graphical approach forms a state transition graph through the agent's interaction with the environment. In such graphs, states act as vertexes and their potential transitions caused by actions are represented as edges. Some efforts form subgoals via directly ranking graph centrality measures of state nodes (e.g., the betweenness centrality [5] and the connection graph stability centrality [14]). The basic idea is that potential subgoals would be special on these measures compared to other vertexes. On the other hand, a more common way is to partition the transition graph as several vertex clusters. States within the same cluster are strongly connected while the intercluster connectivity is minimized. Then border states connecting adjacent clusters can be naturally regarded as subgoals, and options are with the implication of moving from one cluster to another. There are some approaches following this idea but with different implementations. The approach in [15] partitions the transition graph by removing edges with high-value edge betweenness centrality. Meanwhile in [16] the eigenvector centrality is used as a basis to cluster the graph. The authors also present an online option pruning algorithm, attaining substantial performance improvement compared with the betweenness approach and the edge betweenness approach. Another spectral clustering algorithm PCCA+ [17] is also used for skill acquisition [18]. Combining neural network training, it shows effectiveness for complex environments like Atari games. The work presented in [19] finds subgoals in linear time based on forming Strongly Connected Component (SCC) of the graph. What is unique is that this method also exploits historical data to help improve the performance. Additionally, the reformed Label Propagation Algorithm (LPA), a community detection method, is employed to tackle this issue [6]. While LPA has a near-linear time complexity [20], its stability remains doubtful as it can generate redundant communities as well as skills even in simple problems.

One main drawback of the sampling trajectory based approach is that excessive exploration is needed to accurately identify those subgoals. Also, if the goal of the environment changes, previous efforts would be wasted in case that currently detected subgoals do not lead to the new ultimate goal. The graphical approach relies on the transition graph, which would form an understanding on the overall environment. This helps identify potential subgoals no matter what the current goal is. These two branches of approaches do not have a clear border. Actually some approaches can take advantage of both of them, such as the SCC based approach [19]. The approach in [13] also operates on the state transition graph but concentrates more on some metric in the context of the ant colony optimization. The main difference of those efforts lies in how they define the standard for states to be subgoals.

For the graphical approach, it is usually difficult to get a complete transition graph for large state space problems, and hence continuous sampling is necessary to approximate the full view. This requires that the graphical processing can deal with potential increase of new states and new transitions during the exploration. In this paper we propose an option construction and option improvement strategy though incremental community detection. What we concern more is how initially generated options can be updated in online learning. In some related works, the PCCA+ based method [18] calls the cluster algorithm iteratively to get options for large state space problems. Its computation overhead should be expensive. Also, if the resulting partition is quite different from the existing one, the currently formed options can be wasted. The reformed LPA in [6] is extended with an incremental version, but the stability of generated communities is not further discussed. We focus on how detected communities evolve and try to make the option be improved in a stable and efficient way.

## 4. Generating Options from Communities

Constructing options from communities belongs to the graph partition based approach. We first give a brief description of the concept of communities and the process of the Louvain community detection algorithm. Then we describe how options are generated from communities.

### 4.1. Louvain Method for Community Detection

Define *G* = (*V*, *E*) as an undirected unweighted graph where *V* and *E* represent the vertex set and the edge set, respectively. Community detection aims to partition *G* into a finite set of communities *P* = {*C*_1_, *C*_2_,…, *C*_*K*_}, where *V* = *C*_1_ ∪ *C*_2_ ∪ ⋯∪*C*_*K*_ and *C*_*k*1_∩*C*_*k*2_ = *∅* for any distinct *C*_*k*1_, *C*_*k*2_ ∈ *P*. A community is thought as a portion of a graph in which intracommunity edges are dense while intercommunity edges are sparse [21]. The measure* modularity* [22] is often used to evaluate the quality of communities partition:

(5) $$\begin{array}{c} Q\left(\;P\;\right)=\underset{C_{k}\in P}{\sum }\left(\;\frac{m\left(C_{k}\right)}{M}-\frac{d{\left(C_{k}\right)}^{2}}{4M^{2}}\;\right), \end{array}$$

where *m*(*C*_*k*_) is the sum of intracommunity edges of *C*_*k*_, *d*(*C*_*k*_) is the sum of degree of vertexes in *C*_*k*_, and *M* denotes the total number of edges in *G*. The value range of modularity is (−1,1).

Generally higher 𝒬 value means better partitioning; thus the problem of community detection can be solved as seeking for a solution maximizing the modularity. However, because the space of possible partitions grows quite fast, achieving the highest modularity is an NP-hard problem [23]. Algorithms for modularity-based community detection usually try to approximate the maximum of this measure. A comprehensive review on these approaches can be found in [24].

Louvain algorithm [7], which we use in this paper, is a hierarchical greedy optimization approach. It generates communities though iteratively executing a two-phase process. The general procedure is as follows:

1. Initially, each vertex of the graph is assigned to a different community.
2. For each node, check the modularity changes if moving it from its current community to one of neighbor communities, and make the change yielding the positive and maximal modularity increase. The process continues until all nodes are checked, resulting in a first-level partition with local maximal 𝒬.
3. Build a new graph based on the first-level partition, where each node represents a community, and the connected edges are formed with weights as the sum of previous weights of corresponding intercommunity connections.
4. Repeat step (2) and step (3) until no increase in modularity is possible, resulting in the ultimate partition solution.

Louvain algorithm is believed to be one of the fastest modularity-based community detection methods [25]. Assume the graph to be processed has a total of *m* edges and *n* vertexes. The algorithm's runtime complexity is believed to be *O*(*m*). For sparse graphs it is also with a roughly linear growth on *n*. In addition to high efficiency, it can obtain very good-quality results in terms of the modularity measure [25]. The main limitation is its storage demand for large scale networks. Compared to some existing graphical based option generation algorithms, employing Louvain algorithm is of advantage on computation time. For instance, the betweenness centrality computation following [26] should be *O*(*nm*) and *O*(*nm* + *n*^2^log⁡*n*) for unweighted and weighted graph, respectively; LPA grows like *O*(*km*) where *k* is the sum of the algorithm's internal label propagation iterations; and the SCC based method [19] is a linear time algorithm with an *O*(*m* + *n*) complexity.

What should be noted is that, in step (2) of Louvain algorithm, the visit order of vertexes can vary. As indicated in [7], the ordering can influence the computation time as well as the obtained final partition. A default strategy is to traverse nodes in a random order. In [27] the authors evaluate several other vertex ordering strategies and suggest that sort nodes based on descending order of edge degree can bring marginal improvement on computation time than the default strategy. Results in [28] also show that partitions generated by Louvain algorithm following this degree-descending order can have low variance in modularity value (the number in most tested networks is at the level of 10^−5^). In this paper, our implementation uses Louvain algorithm with such ordering strategy by default.

### 4.2. Option Generation from Communities

The main idea of generating options from communities is to form an abstract MDP model on the basis of communities. These communities are converted from the state transition graph of the original problem. State vertexes in the same group can be aggregated as a macrostate, and transitions between macrostates are formed as macroactions (i.e., options). Here we only consider options shifting between two adjacent communities. An option from *C*_*k*1_ to *C*_*k*2_ can be generated by assigning its *ℐ*, *π*_*o*_, and *β*. Specifically,

(i) The initiation states *ℐ* = {*s*_*i*_∣*s*_*i*_ ∈ *C*_*k*1_}.
(ii) The termination condition *β* is defined as
  (6) $$\begin{array}{c} \beta \left(\;s_{i}\;\right)=\left\{\begin{array}{cc} 1, & if\;\;s_{i}\in C_{k2}\\ 0, & otherwise. \end{array}\right. \end{array}$$
The option is expected to stop while the agent reaches the border states of *C*_*k*2_ connecting *C*_*k*1_. In some cases there is more than one state connecting these adjacent clusters, which we can all regard as subgoals.
(iii) The option policy *π*_*o*_ mainly guides the agent moving to *C*_*k*2_. It is assumed that enough episodes of transition experience have been collected before these communities' generation. We adopt the experience replay (ER) mechanism [29] to learn *π*_*o*_ from previous trajectories. ER reuses past experiences to find an optimal policy to reach specified subgoals. During this process, a completion reward in addition to environmental reward signals will be assigned while *β* is satisfied. For nondeterministic transitions, we also set a negative reward if the agent executing an option jumps out of both the source community and the target community.

## 5. Incremental Community Detection for Online Learning

Though Louvain algorithm is computationally efficient and can generate high-quality solutions, it is initially designed for static network analysis. For complex systems sample based methods need to be employed to asymptotically form a satisfying state transition graph. Those initial samples may not be able to reach all states. Thus there is a need to online develop the state transition graph when experience accumulates during learning. As a result, the community detection needs to be performed adapting those dynamic changes. A direct approach is to iteratively execute Louvain algorithm while new states are discovered. However, the algorithm can produce distinct community structures if running multiple times on the same network. Even if we have employed a specific ordering strategy to decrease the variance, partition changes can lead to reconstruction of options, which may waste previous efforts on learning these options' internal policies. Therefore, a trade-off between the optimality and the stability should be considered.

### 5.1. Rule-Based Incremental Community Revision

Here we propose an approach combining the original Louvain algorithm and later incremental processing. Typically Louvain algorithm need be called to create a community assignment from initial samples, and then the incremental processing will work to handle later changes of the transition graph. What we concentrate on is the changes that specifically happen during the learning process, which can be organized as three categories:

 
**Case1**: new vertex addition with edge(s)
 
**Case2**: intracommunity edge addition
 
**Case3**: intercommunity edge addition.

We set rules of how to respond to those cases: For** Case1**, two possible operations can be made, namely, assigning the new vertex to its connected community (Op1) or creating a new community for the vertex (Op2). For** Case2**, intracommunity edge addition actually strengthens the relating community's local modularity measure; hence we can just keep the current community structure unchanged (Op3). For** Case3**, there are also two potential operations to tackle the change, that is, Op3 or merging corresponding communities into a new one (Op4). Selecting a specific operation for a certain case should keep the principle that the modularity of the resulting partition must be the maximal among all choices.

The modularity changes brought by each operation can be deduced from (5). Specifically, we have the following.

*(1) In* ***Case1***. If Op1 is applied to the community structure, the original partition *P* has a new vertex added to one of its communities. Denote the community to be changed as *C*_*i*_, and we have *C*_*i*_ ∈ *P*. The resulting modularity is as

(7) QPop1case1=∑Ck∈PCk≠CimCkM+1−dCk24M+12+mCi+1M+1−dCi+224M+12,

where *P*_op1_^*case*1^ represents the resulting partition after applying Op1 on the original partition *P* in Case1.

The other alternative Op2 creates a new community for the new vertex. Let the new community be *C*_*j*_; then *C*_*i*_ and *C*_*j*_ should be adjacent. Similarly, we have

(8) QPop2case1=∑Ck∈PCk≠CimCkM+1−dCk24M+12+mCiM+1−dCi+124M+12+0−124M+12.

In order to select from Op1 and Op2 in Case1, we can compare their effects on the original partition:

(9) ΔQ1=QPop1case1−QPop2case1=1M+1−dCi−12M+12.

*(2) In* ***Case2***. There is only Op3 as the solution, which just leaves the current partition *P* as is. Define the intracommunity edge being within *C*_*i*_; then we have Δ*d*(*C*_*i*_) = 2, Δ*m*(*C*_*i*_) = 1, and Δ*M* = 1. The modularity for *P*_op3_^*case*2^ is

(10) QPop3case2=∑Ck∈PCk≠CimCkM+1−dCk24M+12+mCi+1M+1−dCi+224M+12.

It can be found that 𝒬(*P*_op3_^*case*2^) has the same form as 𝒬(*P*_op1_^*case*1^), though they result in different partitions. In [30] it has been proved that adding any intracommunity link to a community of a graph will not split it into smaller modules, because this actually increases the community's local modularity. Hence it is reasonable to apply Op3 in response of Case2.

*(3) In* ***Case3***. We suppose the added edge connects *C*_*i*_ and *C*_*j*_. Then for Op3 the modularity becomes

(11) QPop3case3=∑Ck∈PCk≠Ci,CjmCkM+1−dCk24M+12+mCi+mCjM+1−dCi+12+dCj+124M+12.

If Op4 is selected, *C*_*i*_ and *C*_*j*_ are combined into one, which we denote as *C*_*x*_ here. The resulting modularity should be computed as

(12) QPop4case3=∑Ck∈PCk≠Ci,CjmCkM+1−dCk24M+12+mCxM+1−dCi+dCj+224M+12,

where *m*(*C*_*x*_) ≥ *m*(*C*_*i*_) + *m*(*C*_*j*_) + 1 for there may have been already existing intercommunity edges between *C*_*i*_ and *C*_*j*_.

In order to obtain the better operator for Case3, we compare the two as

(13) ΔQ2=QPop4case3−QPop3case3=mCx−mCi−mCjM+1−dCi+1dCj+12M+12.

After the analysis of each operator's effect, we present the incremental processing algorithm to tackle detected state transition changes. As shown in Algorithm 1, the input is a list of changes, with each item corresponding to a specific case. The algorithm is defined to be called after an initial graph being created. Periodically, new nodes and edges on the current transition graph detected from several episodes history will be stored in a list, and then the algorithm will process each item sequentially. Finally a new community partition will be generated, which provides basis for option learning.

The incremental process does not cost too much computational resource. It just associates each graph change with a specific operation. What is more important is the stability it can achieve. We assume that a reasonable portion of the state space is seen before incremental processing. Then the proposed method can keep a high level of stability on existing community partition.

Our implementation draws experience from some researches on dynamic community detection, such as [30, 31]. Their aim is mostly for real-time community tracking, considering vertexes' and edges' addition as well as their removal. Compared to these approaches, our implementation concentrates more on the changes that would happen during online learning.

### 5.2. Option Learning with Evolving Communities

The motivation of employing incremental processing is that, in some complex problems, preliminary sampling may not reach a full view of the state transition graph. As the learning proceeds, new states or new state transitions may occur. The graph keeps evolving, requiring corresponding communities to be updated. In order to tackle this issue, we design the algorithm of option learning with evolving communities as shown in Algorithm 2.

The algorithm contains initial sampling and offline option construction (lines (3)–(10)). It can also be easily embedded in the online learning process. After initial options being constructed, the agent will continue to sample trajectories and periodically check whether option improvement is needed (lines (12)–(21)). The condition of how many samples are required is defined manually according to specific problems. Commonly more than half usually visited states of the target problem should be collected. The incremental processing occasion should be set to ensure every cycle has enough graph changes and the interval is not too long.

We analyze how the incremental community detection will affect current options. For Op1 and Op2, new state vertexes will be incorporated but not change the existing partition, so previous experiences can still be reused. A relatively small number of experiences are enough to regain options internal policies' convergence. Op3 actually does not change the current community assignment. Op4 will merge previous communities, resulting in options with new applicable sets and subgoals. For this occasion ER is required to repeat for more times to learn the policies. But, overall, if enough sampling is performed to construct initial options, Op4 will be rarely used in later processing.

## 6. Experiments and Results Analysis

### 6.1. Four-Room Grid World

**Figure 1:**
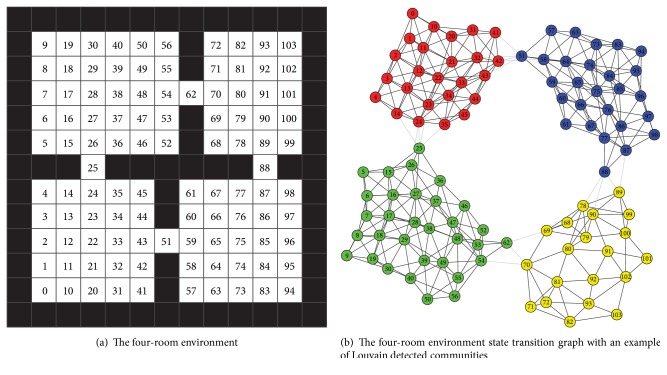
Four-room grid world.

We demonstrate the effectiveness of our presented option construction approach through the benchmark problem, four-room grid world, as depicted in Figure 1(a). The four-room environment contains four adjacent rooms where the agent needs to move from a start point to a specified destination. Four actions are available for the agent, corresponding to moving towards four directions, that is, “East,” “West,” “North,” and “South.” The environment terminates when the agent moves to the destination or a total of 1000 moves are accumulated. The agent receives −1 for every move and +100 if reaching the destination. There is also a probability of 0.1 that a chosen action will not result in moving to its intended direction, but other random ones instead.

Our experiments compare the performance of five different types of agents, that is, the *Q*-primitive agent, the manual-option agent, the Louvain-option agent, the LPA-option agent, and the betweenness-option agent. The *Q*-primitive agent learns with primitive actions following (1). The latter four agents all operate on options and learn using intraoption learning as (3) and (4). The main difference is that the manual-option agent has manually defined options indicating the shift between adjacent rooms with optimal paths. Specifically, we set the goal location in the TopRight room, and four options, including BottomLeft → TopLeft, TopLeft → TopRight, BottomLeft → BottomRight, and BottomRight → TopRight, are defined in the manual-option agent. Meanwhile, the Louvain-option agent and the LPA-option agent employ options constructed by our presented algorithm and the LPA community detection algorithm [20], respectively. Both agents construct the options based on the presampled 10 episodes of experiences. Also, as previously mentioned, these generated options take states in a source community as the initiation set and will get terminated if the current state falls in the target community. The betweenness-option agent is implemented following the approach in [5], where nodes with high-value betweenness centrality measures will be chosen as subgoals. The implementation employs the scoring measure presented in [14] to reinforce local maxima:

(14) $$\begin{array}{c} S_{u}={BC}_{u}{\left(\;\frac{{BC}_{u}}{\max_{v\in N\left(u\right)}{BC}_{v}}\;\right)}^{2}, \end{array}$$

where *u* is a graph node, BC_*u*_ is the betweenness centrality value of *u*, and *N*(*u*) is the neighbor nodes set of *u*. Here we set nodes with top 5%  *S*_*u*_ value as subgoal states. Also, unlike community based options, the betweenness based options take the identified subgoals as termination sets and a certain number of states near subgoals as initiation sets.

The four-room environment has 104 states. It is small and it is easy to collect all of them, so the incremental processing need not be triggered. Some other parameters are set in the same way. In every step, agents choose actions using the *ϵ*-greedy selection strategy with *ϵ* = 0.1. The learning rate *α* = 0.1 and the discount factor *γ* = 0.99. Results are all collected from 50 independent runs. Each run contains totally 100 episodes in which the goal state is kept fixed in the TopRight room while the agent's start position is randomly chosen.

**Figure 2:**
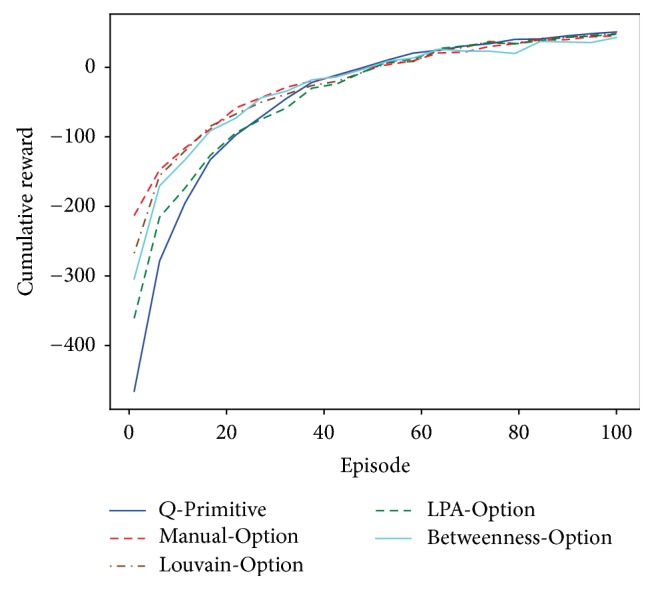
The four-room problem results of comparison with different learning algorithms.

The results are demonstrated in Figure 2. In order to clearly show the performance differences of each algorithm, the curve is drawn from average accumulative reward of every 5 episodes. We can see that the four option learning agents all have better learning speed than the vanilla *Q*-learning at earlier stages. After that they all get converged within 100 episodes. The manual-option agent has generally the best performance among all learning algorithms. This lays a foundation on evaluating the three automatic option generation approaches. The Louvain-option agent does not catch up the manual-option initially. That is mainly because some options generated through Louvain algorithm are redundant for achieving the goal.  Figure 1(b) shows an example of Louvain detected communities on four-room problem's state transition graph. There are 4 clusters, each corresponding to a macrostate. Then a sum of 8 options is generated in terms of macrostate transitions. This gives the agent more available choices during learning, hence more time to reach convergence. The LPA based approach is similar. Further, it is outperformed by the Louvain approach because its generated graph partitions are usually with less quality. The betweenness-option agent has close performance as the Louvain-option agent. In our settings it has 6 subgoals identified during learning, corresponding to 6 options. While this number in the Louvain based approach is mostly 8 or 10.

In our implementation practice, subgoals found by the betweenness-option agent and the Louvain-option agent can both cover the four gate nodes (62, 25, 88, and 51) or at least their neighbors. The performance difference in the four-room environment is mostly derived from how they construct options. Specifically, we found that the initiation set of betweenness based options should contain enough states in order to attain good performance. This ensures that the learning agent has executable options in most states in addition to primitive actions. From this perspective, constructing options based on moving from one community to another may be a more convenient way as there is no need to tune such parameters and detected communities naturally contain all state nodes.

### 6.2. Small-Scale Pac-Man

**Figure 3:**
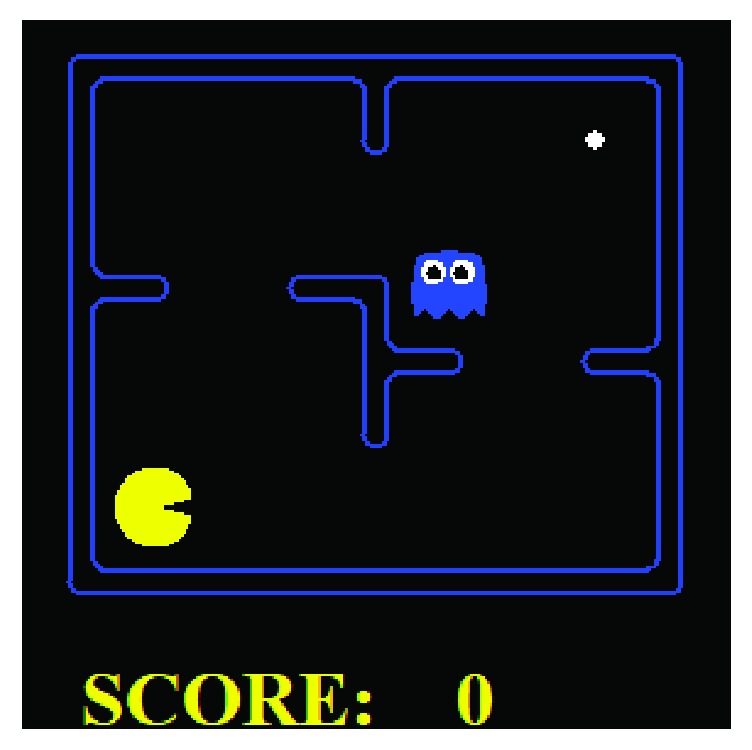
A snap shot of the evaluated small-scale Pac-Man problem. Only one dot and one ghost are set.

The game Pac-Man has been popular for testing AI algorithms. In this environment the Pac-Man agent needs to collect all dots distributed in the map before colliding with some ghost. This section concentrates on a small-scale Pac-Man problem, as shown in Figure 3, where the map is similar to the four-room grid world. It contains only one dot and one ghost. However, it is more complex and uncertain than four-room problem as the ghost position should be considered and the state transition is determined not only by the Pac-Man agent but also by the ghost.

The platform we use for experiments is an open source implementation (http://ai.berkeley.edu) developed by University of California, Berkeley. In its settings, the Pac-Man can move “Up,” “Down,” “Left,” and “Right” and “Stop.” The environment ends if the Pac-Man moves to eat the dot or is eaten by the ghost. As with rewarding signals, the agent receives −1 for every elapsed time, +500 for eating the dot, and −500 if being eaten. The state can be represented as a three-element tuple {pos_pac, pos_ghost, pos_dots}. The first and the second element denote the position of Pac-Man and the ghost, respectively. The last one is the current distributions of dots. Each element can be described as a binary matrix, with the size as MapWidth × MapHeight and the value *v*_*mn*_ indicating the corresponding item's existence (*v*_*mn*_ = 1) or absence (*v*_*mn*_ = 0) in (*m*, *n*) position of the map. As the map has 34 walkable positions in total, the whole state space is 34 × 34 × 2 = 2312.

The main aim of this experiment is to show the performance of learning options with evolving communities. As it is required to consider the position of the ghost, it is not easy to manually define high-quality options for the Pac-Man agent. Here we mainly focus on the comparison of the Louvain-option learning as well as the incremental-option learning. The Louvain-option learning learns with options only generated from initially sampled experiences, while the incremental-option learning includes the option improvement processing as Algorithm 1.

The Pac-Man problem is special for testing the incremental-option learning. Firstly, specific procedures are required to achieve the final goal, which can show the superiority of HRL compared with flat RL. Secondly, its state space is not huge, enabling options to be constructed and improved through the tabular *q*-function representation. Also, there are states or state transitions that would be rarely visited or experienced, and prior sampling cannot form a complete state transition graph. These are all suitable for the incremental processing.

**Figure 4:**
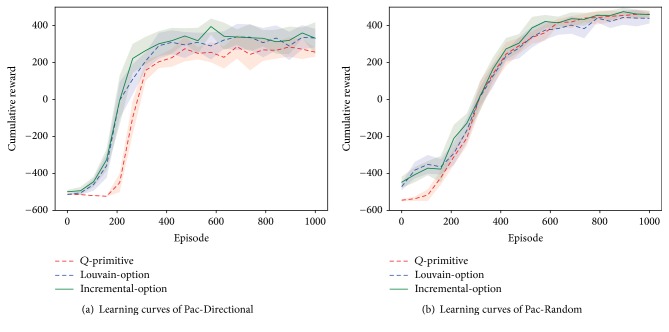
Small-scale Pac-Man problem results. Comparison of the proposed incremental-option learning method with *Q*-learning with primitive actions and Louvain-option learning. Shadow areas denote standard deviation value.

Two different types of ghosts are evaluated in the Pac-Man problem, that is, “DirectionalGhost” and “RandomGhost.” The former rushes to the Pac-Man agent with a probability of 0.8 and otherwise acts randomly, while the latter makes purely random moves. We refer to these two scenarios as “Pac-Directional” and “Pac-Random,” respectively. All agents use *ϵ*-greedy strategy (*ϵ* = 0.05) and learn with *α* = 0.2 and *γ* = 0.8. Each learning experiment lasts for a total of 1000 episodes. For option learning, we first collect 200 episodes of experiences to construct options, which act as the base for Louvain-option learning and incremental-option learning. ER during the incremental processing employs the same set of learning parameters. Results are averaged from 50 independent runs.  Figure 4 shows these results, where average rewards of every 50 episodes are given.

It can be found that in the Pac-Directional scenario the two option learning approaches have faster learning speed than *Q*-primitive. They also achieve higher average scores while being converged. Comparing the two, incremental-option performs slightly better than Louvain-option. At an earlier stage, the two have similar learning speed. While approaching convergence, incremental-option obtains generally higher scores, though such superiority is not very obvious. In the Pac-Random scenario, option learning methods still perform better than *Q*-primitive. However, changes emerge after 400 episodes. Louvain-option starts to slow the same learning speed and even achieve fewer rewards than the converged value of *Q*-primitive. On the contrary, incremental-option keeps a little faster learning speed than *Q*-primitive and finally converges to the same value. Actually, due to the increased uncertainty of state transitions, the three algorithms all become slower in terms of learning speed, though the converged reward is higher than that in the Pac-Directional scenario. The two option learning approaches have the same option base; thus they produce similar results at earlier stages. On the other hand, incremental-option learning continuously processes new experiences to approximate the true state transition graph. So at later stages it would have improved options than Louvain-option, thus obtaining better performance. From these results, we get that Louvain-option learning can have better performance than *Q*-primitive in relatively deterministic environments, while for more complex and uncertain problems it requires incremental-option improvement to be effective.

Take a deeper look into incremental-option learning, we show the sampled state transition graph changes during these experiments in Table 1. The initial amount of nodes and edges is from the 200 episodes experience from which the Louvain-options are created. Those “final amount” data denote corresponding numbers at the end of learning. It is obvious that the initially sampled states and transitions occupy most of those final amounts. They act as basis for initial option sets. Nodes and edges in Pac-Random have greatly more quantities than those in Pac-Directional. The former also has more increased edges in case that its increased nodes are less than the latter. These all demonstrate the dynamics of the Pac-Random scenario. Louvain-option learning shows better performance in the relatively deterministic scenario, while it cannot handle the more uncertain one. That is why the incremental processing is necessary.

**Figure 5:**
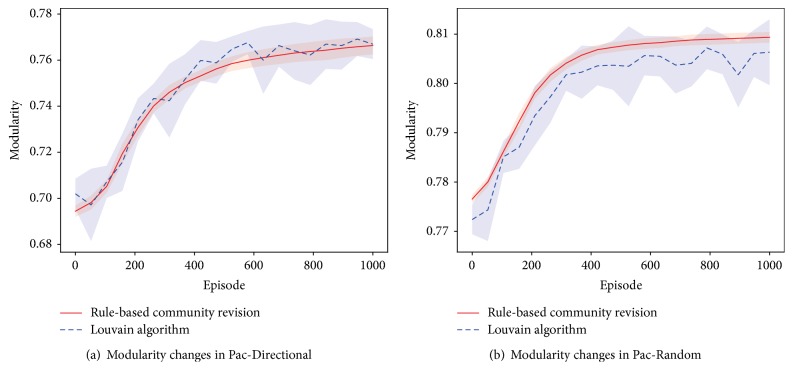
Modularity changes of incremental-option learning in small-scale Pac-Man problem. Comparison of the presented rule-based revision method with iteratively calling Louvain algorithm. Shadow areas denote standard deviation value.

We collect the modularity changes of every 50 episodes during the incremental-option learning process. At each collection, Louvain algorithm is also called on the currently sampled state transition graph. A modularity value is computed from the resulting partition and recorded for comparison.  Figure 5 shows these curves, from which we see that the incremental community revision results in steady increase of the modularity value in both scenarios. Compared to Louvain generated results, our presented approach can have satisfying performance in terms of the modularity value. In Pac-Directional it does not show much inferiority and in Pac-Random it even suppresses calling Louvain alone. What is more important is that it does not lose the stability of graph partitioning. As has been mentioned, even for the same graph, Louvain algorithm would generate different partitions. Our presented incremental community detection approach only operates on part of the communities, keeping remaining unchanged. As incremental-option learning requires options' internal policy to be updated for every community change, such stability ensures that most previously learned *π*_*o*_ can be reused.

## 7. Discussion and Conclusion

This paper proposes an incremental community detection based option construction and option improvement algorithm. The main idea is to create and update options from communities of the sampled state transition graph, which may be evolving overtime. The initial options are constructed from communities generated by Louvain algorithm due to its efficiency and solution quality. The incremental processing is based on doing modifications on existing communities to update options. We first evaluate the quality of options constructed from Louvain detected communities in the four-room environment. Comparative results show that it can accelerate the learning speed compared with flat *Q*-learning. Also, it has similar performance to the betweenness based approach and outperforms the LPA based approach. The performance of incremental-option learning is then tested in the small-scale Pac-Man problem. It is a more complex and uncertain environment where the position of the ghost needs to be considered. We make experiments in two scenarios with different types of ghosts. The results show that learning with Louvain detected options can outperform flat *Q*-learning in the relatively deterministic Pac-Directional scenario. While in the more uncertain Pac-Random, the Louvain based options fail to gain better results. At this time the incremental-option learning still maintains such superiority.

The incremental processing mainly tackles newly encountered transitions during learning, resulting in the addition of edges and nodes to the original sampled graph. The process works by using rule-based revisions to update existing communities, which proves effective in increasing the modularity in our experiments. Compared with using initially generated options alone, the incremental-option learning would update options with consideration of those new state nodes and edges. In complex and uncertain environments, the initially sampled transition graph may not contain all nodes or edges, which would make the generated options not adaptable to the real environments. This can be revealed in the Pac-Random results, where the Louvain-option agent cannot even reach the *Q*-primitive agent at end. On the contrary, the incremental-option agent has the same option base but can achieve higher scores. The underlined reason is that it contains processing of those incremental changes to make the transition graph more complete, thus continuously improving the quality of those options. It does not make too many changes on the originally detected communities but more on small-scale compensations of them. So the performances gained by the two option learning approaches do not differ much. However, these small compensations bring help, showing that the presented incremental processing is effective and necessary.

One main limitation of our current approach is that the sampled graph is unweighted and undirected. This to some extent simplifies the learning environment. Using weighted and directed graph should be more suitable, as state transitions are direct and there exists a probability for each transition. Continuously approximating the edge weights relies on exploiting historical information. This is similar to the SCC based method [19] which takes advantage of both graphical approach and frequency based approach. The expected advantage is to help locating subgoals more accurately. On the other hand, the unweighted undirected graph used in our experiments can also give a rough description on the corresponding problem. So our approach still shows superiority to flat *Q*-learning.

Further, the graph partition based approach constructs options from the ground state space, which may be less effective for some complex problems. Some other problem decomposition techniques can provide insights into this issue. For instance, the approach in [32] employs the* subspace generalization* to increase the learning speed. Here the subspace is a subdimension of the original multidimensional state space. The main idea is to exploit subspace policies gained from early experiences and also to avoid their excessive use. This is particularly effective for problems where subspaces are more informative. Like in the Pac-Man environment, some states may be rarely encountered while their corresponding representation in subspaces (such as the Pac-Man agent's position) has been experienced frequently. Combining skill acquisition with subspace generalization should bring performance improvement for such problems. Another kind of technique in [33] forms MDP abstractions through symbolic descriptions. The authors also present a framework to combine option discovery and symbolic representation for creating multilevel abstraction hierarchies. This provides a novel use of options, which enables fast high-level planning over ground state representation. As their work focuses more on the planning process, more investigations are required to automatically construct the presented abstraction hierarchy.

Our current implementation has shown effectiveness in some simple grid world environments. Some recent researches on automatic skill acquisition tend to solve more complex problems. For instance, the work in [34] presents a skill based transfer learning framework for continuous domains. The authors map the actual state to the discrete domain and also employ the graphical approach to generate options. Additionally, the emergence of deep learning techniques has inspired several efforts to combine the option framework with deep neutral networks (such as [35, 36]), which all showed better performance than regular deep *Q*-learning. These approaches form options from different perspectives but to some extent draw experiences from those graphical methods. Our approach, however, is potential to be extended in such context. Another aspect is that, as a form of skill, constructing options is sometimes unnecessary to consider the whole state space. For example, in [37], to solve the complex navigation tasks of the* Infinite Mario* game, the local movement strategy is represented as options trained with only the Mario-agent surrounded by partial states. Then the solution for the whole game is based on planning with these local skills. As it is often possible to get good-quality options in small state spaces, combining planning with those locally effective options can be an effective way to solve problems with large state spaces.

For future works, we will explore constructing options from weighted directed graphs, which may better handle nondeterministic environments. How to perform incremental processing on this type should be considered. Also, there is a need to better schedule the experience replay in order to get more efficient option internal policy convergence. We would also like to explore combining the presented incremental-option learning with other techniques to handle more complex real-world problems.

## Figures and Tables

**Algorithm 1:**
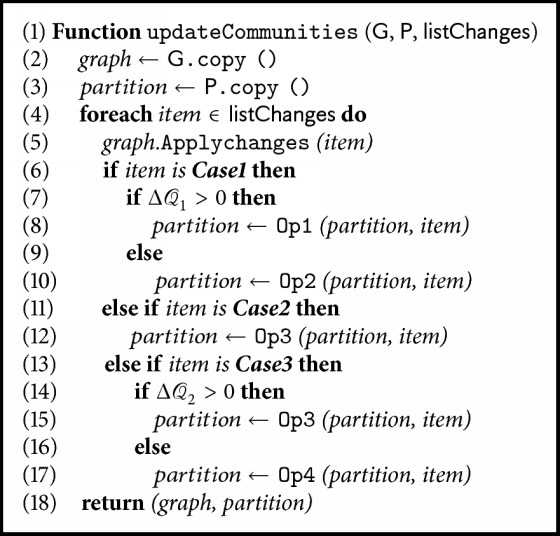
Incremental processing algorithm for transition graph changes.

**Algorithm 2:**
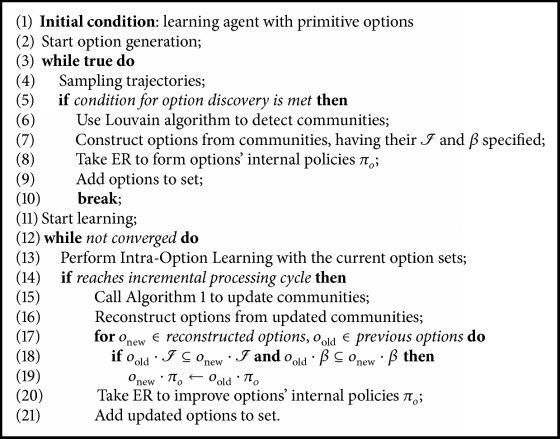
Option learning with evolving communities.

**Table 1:** Sampled state transition graph changes during learning.

Scenario	Pac-Directional	Pac-Random
Nodes		
Initial amount	561	967
Final amount	839.5 (25.4)	1105.4 (11.1)
Increased amount	278.5 (25.4)	138.4 (11.1)

Edges		
Initial amount	1504	3406
Final amount	2368.1 (60.8)	4331.6 (16.5)
Increased amount	864.1 (60.8)	925.6 (16.5)

## References

[1] Parr R., Russell S. J., Jordan M. I., Kearns M. J., Solla S. A. Reinforcement learning with hierarchies of machines.

[2] Sutton R. S., Precup D., Singh S. (1999). Between mdps and semi-mdps: A framework for temporal abstraction in reinforcement learning. *Artificial Intelligence*.

[3] Dietterich T. G. (2000). Hierarchical reinforcement learning with the MAXQ value function decomposition. *Journal of Artificial Intelligence Research*.

[4] McGovern A., Barto A. G., Brodley C. E., Danyluk A. P. Automatic discovery of subgoals in reinforcement learning using diverse density.

[5] Simsek Ö., Barto A. G., Koller D., Schuurmans D., Bengio Y., Bottou L. Skill characterization based on betweenness.

[6] Bacon P.-L., Precup D. Using label propagation for learning temporally abstract actions in reinforcement learning.

[7] Blondel V. D., Guillaume J., Lambiotte R., Lefebvre E. (2008). Fast unfolding of communities in large networks. *Journal of Statistical Mechanics: Theory and Experiment*.

[8] Sutton R. S., Barto A. G. (1998). *Reinforcement Learning - An Introduction*.

[9] Watkins C. J. C. H. (1989). *Learning from Delayed Rewards*.

[10] Sutton R. S., Precup D., Singh S. P., Shavlik J. W. Intra-option learning about temporally abstract actions.

[11] Simsek Ö., Barto A. G., Brodley C. E. Using relative novelty to identify useful temporal abstractions in reinforcement learning.

[12] Demir A., Çilden E., Polat F., Frasconi P., Landwehr N., Manco G., Vreeken J. Local roots: A tree-based subgoal discovery method to accelerate reinforcement learning.

[13] Ghafoorian M., Taghizadeh N., Beigy H. Automatic abstraction in reinforcement learning using ant system algorithm.

[14] Ajdari Rad A., Hasler M., Moradi P. Automatic skill acquisition in reinforcement learning using connection graph stability centrality.

[15] Davoodabadi M., Beigy H., Prasad B., Lingras P., Nevatia R. A new method for discovering subgoals and constructing options in reinforcement learning.

[16] Taghizadeh N., Beigy H. (2013). A novel graphical approach to automatic abstraction in reinforcement learning. *Robotics and Autonomous Systems*.

[17] Weber M., Rungsarityotin W., Schliep A. (2004). *Perron cluster analysis and its connection to graph partitioning for noisy data*.

[18] Lakshminarayanan A. S., Krishnamurthy R., Kumar P., Ravindran B. Option discovery in hierarchical reinforcement learning using spatio-temporal clustering. https://arxiv.org/abs/1605.05359.

[19] Kazemitabar S. J., Taghizadeh N., Beigy H. (2017). A graph-theoretic approach toward autonomous skill acquisition in reinforcement learning. *Evolving Systems*.

[20] Raghavan U. N., Albert R., Kumara S. (2007). Near linear time algorithm to detect community structures in large-scale networks. *Physical Review E: Statistical, Nonlinear, and Soft Matter Physics*.

[21] Newman M. (2010). *Networks: An Introduction*.

[22] Newman M. E. J., Girvan M. (2004). Finding and evaluating community structure in networks. *Physical Review E: Statistical, Nonlinear, and Soft Matter Physics*.

[23] Brandes U., Delling D., Gaertler M. Maximizing modularity is hard. https://arxiv.org/abs/physics/0608255.

[24] Fortunato S. (2010). Community detection in graphs. *Physics Reports*.

[25] Lancichinetti A., Fortunato S. (2009). Community detection algorithms: a comparative analysis. *Physical Review E: Statistical, Nonlinear, and Soft Matter Physics*.

[26] Brandes U. (2001). A faster algorithm for betweenness centrality. *Journal of Mathematical Sociology*.

[27] Wickramaarachchi C., Frincu M., Small P., Prasanna V. K. Fast parallel algorithm for unfolding of communities in large graphs.

[28] Chakraborty T., Srinivasan S., Ganguly N., Bhowmick S., Mukherjee A. (2013). Constant communities in complex networks. *Scientific Reports*.

[29] Lin L.-J. (1992). Self-improving reactive agents based on reinforcement learning, planning and teaching. *Machine Learning*.

[30] Nguyen N. P., Dinh T. N., Xuan Y., Thai M. T. Adaptive algorithms for detecting community structure in dynamic social networks.

[31] Shang J., Liu L., Xie F. (2012). A real-time detecting algorithm for tracking community structure of dynamic networks. *6th SNA-KDD Workshop*.

[32] Hashemzadeh M., Hosseini R., Ahmadabadi M. N. (2017). Exploiting generalization in the subspaces for faster model-based learning. *CoRR*.

[33] Konidaris G., Kambhampati S. Constructing abstraction hierarchies using a skill-symbol loop.

[34] Shoeleh F., Asadpour M. (2017). Graph based skill acquisition and transfer Learning for continuous reinforcement learning domains. *Pattern Recognition Letters*.

[35] Bacon P., Harb J., Precup D., Singh S. P., Markovitch S. The option-critic architecture.

[36] Machado M. C., Bellemare M. G., Bowling M. H., Precup D., Teh Y. W. A laplacian framework for option discovery in reinforcement learning.

[37] Dann M., Zambetta F., Thangarajah J. (2017). Integrating Skills and Simulation to Solve Complex Navigation Tasks in Infinite Mario. *IEEE Transactions on Computational Intelligence and AI in Games*.

